# Shannon diversity index: a call to replace the original Shannon’s formula with unbiased estimator in the population genetics studies

**DOI:** 10.7717/peerj.9391

**Published:** 2020-06-29

**Authors:** Maciej K. Konopiński

**Affiliations:** Institute of Nature Conservation, Polish Academy of Sciences, Kraków, Poland

**Keywords:** Genetic diversity, Shannon index, Coalescent simulations, Measures of genetic variation, Sample size effect, Statistical genetics

## Abstract

**Background:**

The Shannon diversity index has been widely used in population genetics studies. Recently, it was proposed as a unifying measure of diversity at different levels—from genes and populations to whole species and ecosystems. The index, however, was proven to be negatively biased at small sample sizes. Modifications to the original Shannon’s formula have been proposed to obtain an unbiased estimator.

**Methods:**

In this study, the performance of four different estimators of Shannon index—the original Shannon’s formula and those of Zahl, Chao and Shen and Chao et al.—was tested on simulated microsatellite data. Both the simulation and analysis of the results were performed in the R language environment. A new R function was created for the calculation of all four indices from the genind data format.

**Results:**

Sample size dependence was detected in all the estimators analysed; however, the deviation from parametric values was substantially smaller in the derived measures than in the original Shannon’s formula. Error rate was negatively associated with population heterozygosity. Comparisons among loci showed that fast-mutating loci were less affected by the error, except for the original Shannon’s estimator which, in the smallest sample, was more strongly affected by loci with a higher number of alleles. The Zahl and Chao et al. estimators performed notably better than the original Shannon’s formula.

**Conclusion:**

The results of this study show that the original Shannon index should no longer be used as a measure of genetic diversity and should be replaced by Zahl’s unbiased estimator.

## Introduction

The Shannon diversity index (Shannon, 1948), also known as the Shannon-Wiener index, Shannon entropy or, incorrectly, the Shannon-Weaver index (Spellerberg & Fedor, 2003), has been used to estimate genetic diversity in numerous studies. It can be utilised to describe variation at multiple levels of genetic organisation from single nucleotide polymorphisms (SNP), through whole species or larger taxonomic units to ecosystems. Due to its additive properties (Jost, 2007), the Shannon index has recently been postulated as a unifying measure for the partitioning of diversity at those levels (Gaggiotti et al., 2018; Sherwin, 2018). Additionally, Sherwin et al. (2017) showed its potential utility in genomic studies. Several population genetics programs and *R* packages calculate Shannon’s *H,* e.g., GenAlEx (Peakall & Smouse, 2012), *DartR* (Gruber et al., 2018), *SpadeR* (Chao, Ma & Chiu, 2016), *vegan* (Oksanen et al., 2019), *poppr* (Kamvar, Tabima & Grünwald, 2014), GenoDive (Meirmans & Tienderen, 2004), *HierDpart* (Qin, 2019) and the index is still in use (e.g.,  O’Reilly et al., 2018; Zhang et al., 2019; Chang, Huang & Liao, 2019).

The measure was initially developed within information theory (Shannon, 1948) but it was soon adopted in studies on species diversity (e.g.,  Good, 1953; Margalef, 1957; Crowell, 1961) and in population genetics (Jain et al., 1975). In principle, Shannon’s *H* takes into account the proportion of each species in an ecosystem studied; hence, it gives a better description of an ecosystem’s diversity than a plain number of species. When the number of species is equal in two locations, the index is capable of distinguishing between sites dominated by a single or only a few predominant species and those where each species has comparable input to the whole biodiversity (Margalef, 1957). Similarly, in population genetics studies, Shannon’s *H* allows distinguishing the level of variation between populations with the same number of alleles, when in some populations loci are dominated by only a few common alleles while in others variation is contributed more evenly by all alleles. The Shannon index is more sensitive to the loss of rare variants (e.g., due to genetic bottlenecks) than heterozygosity, and more informative than allelic richness or a plain number of alleles (Sherwin et al., 2017).

In the original formula of the Shannon index developed within information theory, it is assumed a researcher is capable of counting all words or letters in a text studied. In biological studies, however, researchers depend on a sample from the population and use it as a proxy for the population parameters. The index changes rapidly when the number of low-frequency occurrences grows, while their number depends on the sample size (Basharin, 1959; Chao & Shen, 2003). The probability that all the alleles are sampled falls dramatically when the sample size is small. At the same time, in small samples, the lack of some allele inflates the frequencies of the alleles that have been sampled. As a solution to that, a few unbiased estimators of *H* have been proposed. The methods use jack-knifing (Zahl, 1977), rarefaction (Chao & Shen, 2003) or the Good-Turing frequency formula (Good, 1953; Chao, Wang & Jost, 2013) to account for unsampled components of the system (i.e., species or alleles). Although the issue of sample size in population genetics has been addressed in several studies (e.g.,  Marquez-Sanchez & Hallauer, 1970; Gorman & Renzi, 1979; Chakraborty, 1992; El Mousadik & Petit, 1996; Leberg, 2002; Pruett & Winker, 2008), the dependence of Shannon’s diversity estimation on sample size has never been thoroughly discussed with regard to genetic data. Bashalkhanov, Pandey & Rajora (2009) noticed an increase in the deviation of Shannon’s *H* at small sample sizes; however, the only solution they suggested was increasing the sample size to 60–90 genotypes. However, Sherwin et al. (2017) pointed out that although methods for sampling correction of *H* exist, unbiased estimators remain rarely applied in population genetics studies.

The aim of this study was to assess the effect of sample size and locus properties (mutation rate and the maximum possible number of allelic states) on the estimation of Shannon’s original index (*H*_*MLE*_) and its three unbiased estimators proposed by (Zahl, 1977) (*H*_*Z*_), (Chao & Shen, 2003) (*H*_*CS*_), (Chao, Wang & Jost, 2013) (*H*_*Chao*_). The performance of the four indices was tested extensively on data generated using coalescent simulations. The relative effects of sample size, locus properties and population diversity were analysed with a Generalised Linear Model. A wrapper R function was written to allow for estimation of the four indices directly on *adegenet*’s ‘genind’ objects.

## Materials & Methods

Analyses were conducted in R 3.6.2 (R Development Core Team, 2009). Coalescent simulations as implemented in *fastsimcoal2* ver. 2.6 (Excoffier et al., 2013) were used to generate populations differing in levels of genetic variation due to their demographic histories. The program was called from within the R environment using the *strataG* package ver. 2.0.2 command *fastsimcoal* (Archer, Adams & Schneiders, 2017). Twenty-four microsatellite loci with four different mutation rates (0.0001, 0.0002, 0.0005 and 0.001 mutations per generation) and six different maximum numbers of alleles (3, 6, 9, 12, 15 and 20 alleles) were simulated (Table 1). The model assumed a large population of 10,000 diploid individuals divided into four populations containing 10,000 individuals each. Three of those underwent bottlenecks of different sizes (20, 50 and 500 individuals in populations *P*_20_, *P*_50_ and *P*_500_ respectively) while the fourth, the control population (*P*_*C*_ remained at a stable size of 10,000 individuals. Each of the bottlenecked populations after 20 generations recovered to the original size of 10,000 and was simulated for another 20 generations until time T0 when samples equalling the whole populations were saved both from bottlenecked and control populations. Reference parametric values of four different Shannon’s estimators were calculated for each total population sample:

**Table 1:** Combination of mutation rates and a maximum number of allelic states in the 24 loci simulated in *fastsimcoal2*.

		Max. number of alleles					
		3	6	9	12	15	20
Mutation rate	0.0001	L01	L02	L03	L04	L05	L06
	0.0002	L07	L08	L09	L10	L11	L12
	0.0005	L13	L14	L15	L16	L17	L18
	0.001	L19	L20	L21	L22	L23	L24

- *H*_*MLE*_—the maximum likelihood estimate of *H* based on the original Shannon’s formula (Shannon, 1948, Theorem 2)—probably the most widely used in population genetics;
- *H*_*CS*_—unbiased estimator proposed by Chao & Shen (2003, equation 8);
- *H*_*Chao*_—unbiased estimator proposed by Chao, Wang & Jost (2013, equation 7);
- *H*_*Z*_—jackknife estimate proposed by Zahl (1977, equation 7).

Additionally, expected heterozygosity (*He*) was calculated as a reference measure of the genetic variation of the total populations.

The four *H* estimators are calculated using the function *Diversity* in R package *SpadeR* (Chao, Ma & Chiu, 2016). Initial attempts to apply the function to the simulated set showed that the function often fails due to a problem with another nested function that estimates species richness. Moreover, the function has to be run separately for each locus to obtain locus-specific *H*. Therefore, a generic function (*ShannonGen*) was written using the formulas from the *shannon_index* function nested in *Diversity*. The function takes *genind* objects as the input and transforms them into abundance data, which is required by functions taken from *shannon_index*. *ShannonGen* returns a list containing user-selected estimators of *H* for all loci and populations included in the input object. The function can be acquired from GitHub (https://github.com/konopinski/Shannon/).

The Shannon diversity index estimators were calculated for samples of *Ns* = 5, 20, 80 and 200 genotypes drawn randomly without replacement from the simulated populations, The numbers of samples used were selected to represent four sampling scenarios:

- limited availability of samples—a situation often faced in studies on rare or elusive animals—*Ns* = 5 samples;
- a minimum acceptable number of samples as suggested by Pruett & Winker (2008) commonly occurring in population genetics studies—*Ns* = 20 samples;
- optimal sampling, according to Bashalkhanov, Pandey & Rajora (2009)—*Ns* = 80 samples.
- a very large sample with presumably low sampling error—*Ns* = 200 samples.

Additionally, the parametric values were obtained from the simulated populations.

Sampling variance of each *H* estimator was assessed using 500 sets of samples randomly drawn from each of the simulated populations. The standard deviations (*SD*) of the results were calculated for each sample size and demographic scenario. The *SD* values distributions were compared pairwise between metrics. The level of significance was assessed based on 100,000 comparisons of the randomly drawn pairs of the *SD* values.

For each *H* estimator, a relative bias was calculated as *rB* = (($\hat {H}-H$)/*H*), where $\hat {H}$ is the estimate of a given metric in a sample, and *H* is the parametric value of a given estimator. The bias was estimated only once per each metric/population/iteration (i.e., 16,000 times). The error of the metric was estimated as a mean relative squared error (MRSE):

$MRSE= \frac{1}{500} {\mathop{\sum }\nolimits }_{i=1}^{500} \frac{{ \left( {\hat {H}}_{i}-H \right) }^{2}}{H} $, where $\hat {H}$_*i*_ is an estimate of a metric in the *i*-th sample of the 500 resamplings that were carried out to estimate the mean.

To explore the factors influencing the errors of the analysed indices, the generalised linear model, *glm*, a function from R’s *stats* package, was used. The model included *MRSE* as the response and four explanatory variables: *H* estimator, sample size, population gene diversity and locus. To avoid overparametrisation, the GLM analyses were performed in two steps. Firstly, *MRSE* of mean *H* values over the 24 simulated loci were provided to the model as an effect; secondly, *MRSE* calculated for each locus separately was used.

Because the relation between sample size and *MRSE* is asymptotic, and the effect size may depend on arbitrarily selected number of samples, the values were provided to the model as categorical values (factors). The best-fitting model was selected based on the Akaike Information Criterion (AICc) as implemented in the *model.sel* function from the R package *MuMIn* (Bartoń, 2019). The GLM results were analysed using the *Anova* function from the package *car* (Fox & Weisberg, 2019). The effects of the explanatory variables were visualised using the R package *effects* (Fox & Weisberg, 2018; Fox & Weisberg, 2019). Tukey’s Honest Significant Difference (Tukey’s HSD; Tukey, 1977) method was used to test whether the differences between the factors were significant. The function *glht* from the R package *multcomp* (Hothorn, Bretz & Westfall, 2008) was used to perform the analysis, while *cld* was used to summarise results and present them as compact letter displays (Piepho, 2004). The script used for simulations is deposited at Github (https://github.com/konopinski/Shannon/).

## Results

Each metric was estimated altogether 192 096 000 times in 24 loci, 4 populations (*P*_*C*_, *P*_500_, *P*_50_, *P*_20_), 5 sample sizes (i.e., 5, 20, 80 and 200 genotypes and for the whole simulated population to obtain parametric values), 500 randomizations and 1000 simulation repetitions. Mean expected heterozygosities calculated for the simulated total populations ranged from *He* = 0.318 to *He* = 0.789 with median *He* = 0.683. The parametric values of the four *H* indices were similar within each of the simulated demographic scenario both in terms of their median values and their ranges (Table 2).

**Table 2:** Summary of the simulations. Minimum, maximum and median values of the *H* estimators and the Nei’s gene diversity (*Hs*) calculated for the four simulated demographic scenarios.

Population		*H*_*MLE*_**	*H*_*Z*_**	*H*_*CS*_**	*H*_*Chao*_**	*Hs*
	min	1.5159	1.5161	1.5160	1.5161	0.6996
*P*_*C*_**	median	1.6706	1.6708	1.6706	1.6708	0.7568
	max	1.7834	1.7836	1.7834	1.7836	0.7889
	min	1.4220	1.4221	1.4221	1.4221	0.6764
*P*_500_**	median	1.6075	1.6077	1.6075	1.6077	0.7428
	max	1.7409	1.7411	1.7409	1.7411	0.7795
	min	0.9940	0.9941	0.9941	0.9941	0.5347
*P*_50_**	median	1.2007	1.2009	1.2008	1.2009	0.6266
	max	1.3441	1.3443	1.3442	1.3443	0.6843
	min	0.5927	0.5928	0.5929	0.5928	0.3179
*P*_20_**	median	0.8281	0.8282	0.8282	0.8282	0.4731
	max	1.0077	1.0079	1.0079	1.0079	0.5734

Attempts to estimate the sampling variance of *H*_*CS*_ failed in 1,689 out of 16,000 resampling attempts, i.e., roughly 10%. The problem occurred only in the smallest simulated samples (*Ns* = 5) and only in the most variable populations: 937 in *P*_*C*_ and 752 in *P*_500_. Similarly, mean *H*_*CS*_ could not be estimated in 10 cases in the 16 000 population sampling simulations extracted from the whole set to estimate bias. The problem occurred only in the most variable populations (7 failures in *P*_*C*_ and 3 in *P*_500_) and in the smallest sample size. The loci that caused the problem were those simulated with a large number of possible allelic states (12, 15 and 20 alleles) and the problem was more frequent in the loci with higher mutation rates (Table S1). Due to the large proportion of missing data, the estimates of *H*_*CS*_ from the populations *P*_*C*_ and *P*_500_ simulated with the smallest sample size *Ns = 5* were excluded from the sampling variance comparisons, and the 10 samples that failed at estimation of *H*_*CS*_ in the simulations of population sampling were excluded from assessment of the performance of the *H* indices.

**Figure 1:**
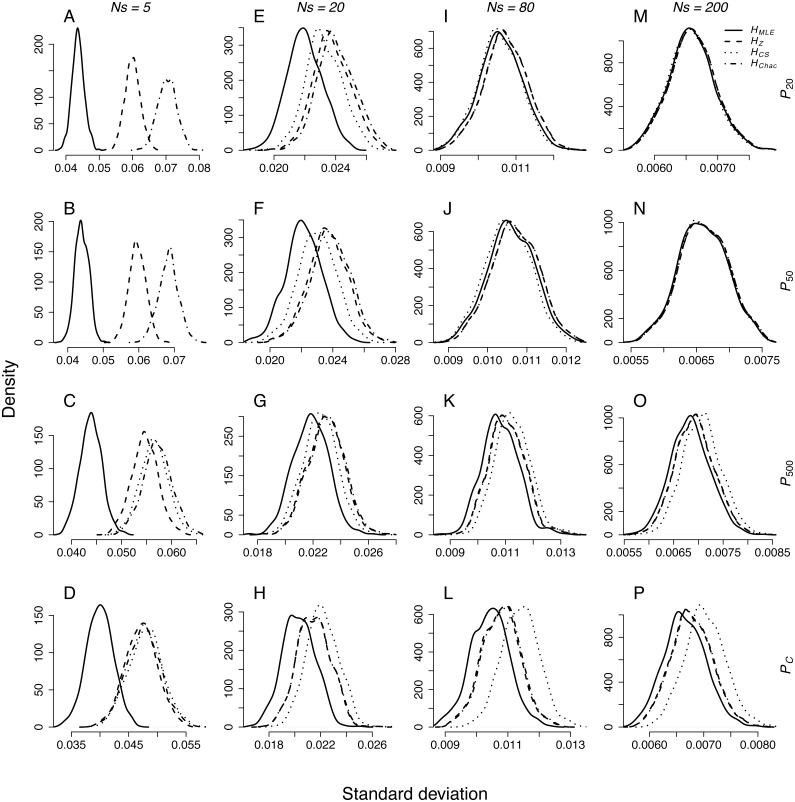
Density plots of SD distributions obtained through repeated sampling of *Ns* = 5, 20, 80 and 200 genotypes from each population representing different demographic scenario in each simulation. Sample sizes (columns): (A–D) *Ns* = 5, (E–H) *Ns* = 20, (I–L) *Ns* = 80, (M–P) *Ns* = 200. Demographic scenarios (rows): (A), (E), (I), (M) *P*_20_, (B), (F), (J), (N) *P*_50_, (C), (G) ,(K), (O) *P*_500_, (D), (H), (L), (P) *P*_*C*_.

The standard deviation of the results distribution from the repeated sampling depended on sample size, demographic scenario and *H* estimator (Fig. 1). Standard deviations of *H*_*MLE*_ were significantly lower in all pairwise comparisons (Table 3). Among the remaining metrics, the estimates of *H*_*Z*_ had significantly narrower distribution then *H*_*Chao*_ in control populations (*P*_*C*_) and the populations that underwent a bottleneck of 500 genotypes (*P*_500_). In larger sample sizes, *Ns* = 20, 80 and 200, the distributions of standard deviations of *H*_*Z*_, *H*_*Chao*_ and *H*_*CS*_ were not significantly different in any of the pairwise comparisons.

**Figure 2:**
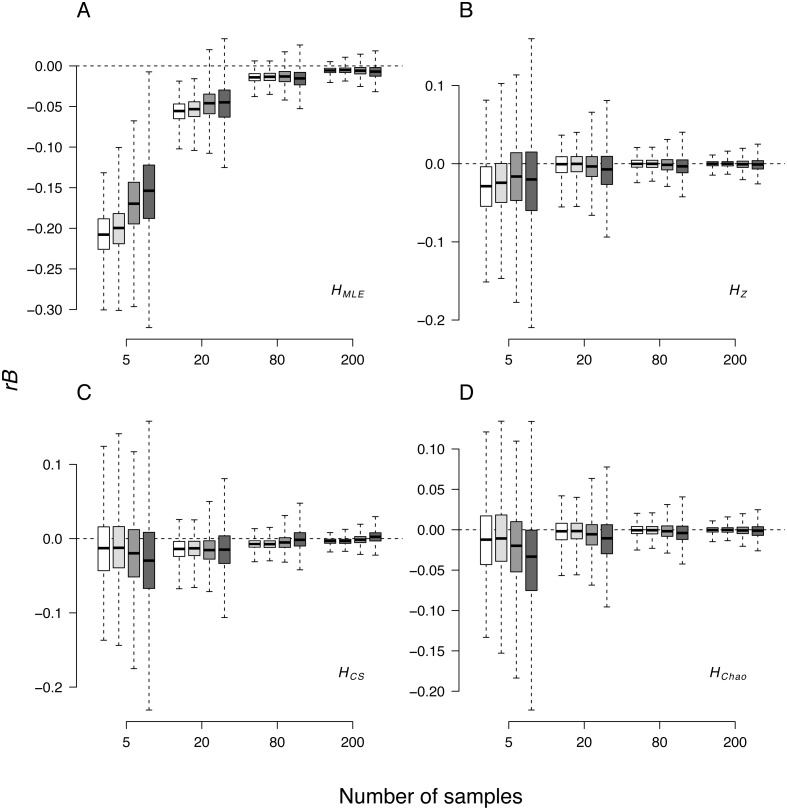
Box-whisker plot of relative bias (*rB*) of the four Shannon *H* estimators in all the simulated demographic scenarios and sample sizes. (A) *H*_*MLE*_, (B) *H*_*MLE*_, (C) *H*_*CS*_, (D) *H*_*Chao*_. The whiskers represent the range of maximum and minimum values, the top and bottom of the boxes represent the 75% and 25% quartiles. White—non-bottlenecked, control population (*P*_*C*_), light-grey—bottleneck of 500 individuals (*P*_500_), dark-grey—bottleneck of 50 individuals (*P*_50_), anthracite—bottleneck of 20 individuals (*P*_20_).

The median relative bias (*rB*) of the *H* estimates averaged over the 24 loci was inversely associated with the sample size in all cases (Fig. 2, Table 4). As compared to other *H* estimators at all sample sizes, the strongest negative departure from parametric values (i.e., calculated from the total population) was observed in *H*_*MLE*_ estimates. Among the remaining three *H* estimates, *H*_*Chao*_ and *H*_*Z*_ were the least biased (Table 4). Except for the *H*_*MLE*_ estimates at sample sizes below 80 genotypes, 95% confidence intervals of *H* estimators always spanned the parametric value of the simulated data. The observed relative bias ranges were markedly wider in the smallest samples.

**Figure 3:**
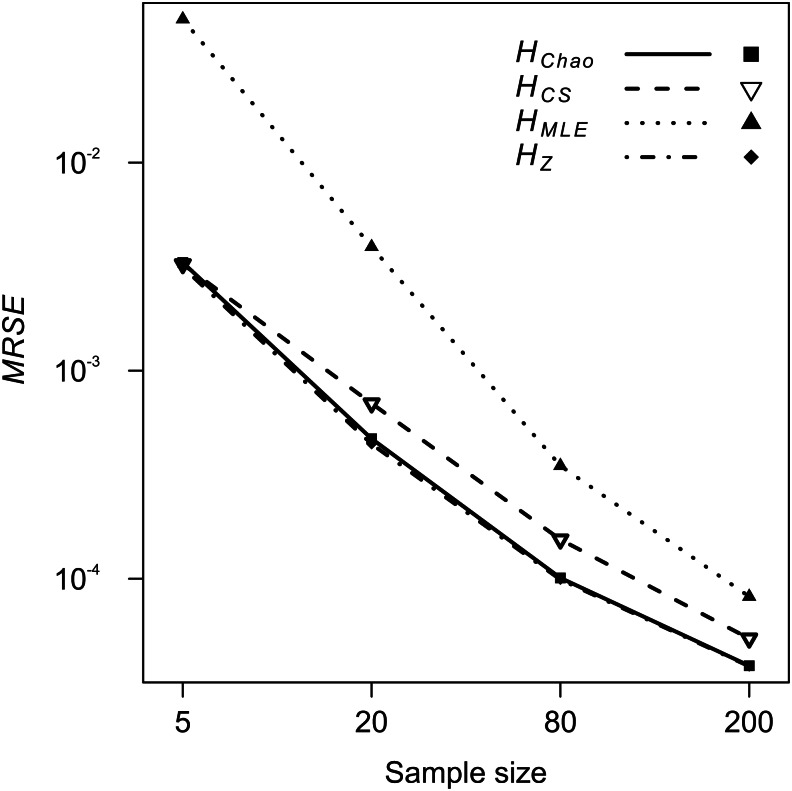
GLM results: the effect of the sample size predictor on the mean relative squared error (*MRSE*) of the four Shannon *H* estimators.

**Figure 4:**
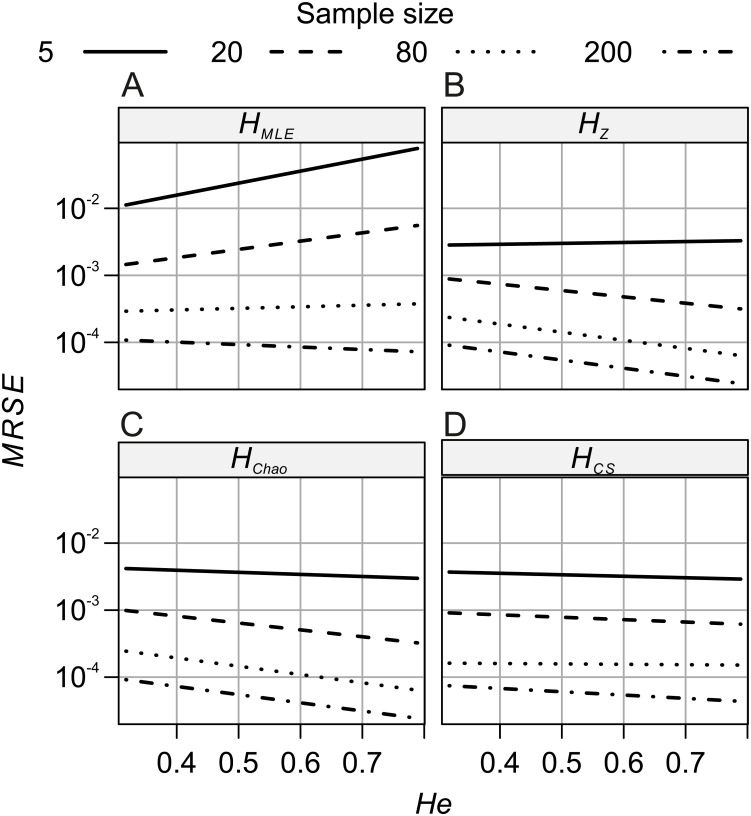
GLM results: the effects of total population’s expected heterozygosity (*He*) and the sample size (*Ns*) predictors on the mean relative squared error (*MRSE*) of (A) *H*_*MLE*_, (B) *H*_*Z*_, (C) *H*_*CS*_ and (D) *H*_*Ch*_.

The analyses of relative error (*MRSE*) provided similar findings. Based on AICc summarised by MuMIn function, the gamma distribution of *MRSE* with a log link function was used in the GLM. According to ANOVA test of the GLM results, all four factors—locus, metric, sample size and expected heterozygosity of the total population (*He*)—were significantly associated with the error levels (*p* = 10^−15^). The median *MRSE* of *H* estimators was negatively associated with the sample size. When compared to *Ns* = 200 genotypes, the slope of the relation, *β*, increased on decreasing sample size, from *β* = 0.97 for *Ns* = 80 genotypes, to *β* = 4.46 for *Ns* = 5 individuals (*p* = 10^−15^). Tukey’s HSD analysis of GLM results confirmed the differences between error levels among all the different sample sizes were significant for all metrics. The strongest effect of sample size on *MRSE* was observed for *H*_*MLE*_ (Fig. 3). The remaining *H* estimators were markedly less affected by a small sample size with *H*_*CS*_ performing slightly worse than *H*_*Z*_ and *H*_*Chao*_. Analysis of GLM results using Tukey’s HSD showed that among all the metrics, *H*_*Chao*_ and *H*_*Z*_ were significantly less affected by error than the other two estimators at the majority of sample sizes (Table 5). Only at the smallest sample size, the difference between *H*_*Chao*_, *H*_*Z*_ and *H*_*CS*_ was not significant. The error levels were also strongly negatively associated with the *He* of the total population (*β* = −0.71, *p* = 10^−15^, Fig. 4). In the case of *H*_*MLE*_ and *H*_*Z*_, the effect depended on the sample size, being, positive at the smaller sample sizes: *Ns* ≤ 80 in *H*_*MLE*_ and Ns = 5 in *H*_*Z*_.

**Table 3:** Sampling variance of the *H* estimators calculated for *Ns*= 5 genotypes. Mean *SD* of each estimator in each demographic scenario, and the *p*-values of pairwise comparisons of the *SD* distributions.

Population	Metric	Mean SD of metric	*p*-values		
			*H*_*Z*_**	*H*_*CS*_**	*H*_*Chao*_**
*P*_*C*_**	*H*_*MLE*_**	0.0436	10^−5^	–	10^−5^
	*H*_*Z*_**	0.0599		–	0.0038
	*H*_*CS*_**	–			–
	*H*_*Chao*_**	0.0704			
**	**				
*P*_500_**	*H*_*MLE*_**	0.0439	10^−5^	–	10^−5^
	*H*_*Z*_**	0.0596		–	0.0191
	*H*_*CS*_**	–			–
	*H*_*Chao*_**	0.0683			
**	**				
*P*_50_**	*H*_*MLE*_**	0.0439	0.0001	0.0004	0.0016
	*H*_*Z*_**	0.0547		0.6397	0.5189
	*H*_*CS*_**	0.0565			0.8547
	*H*_*Chao*_**	0.0572			
**	**				
*P*_20_**	*H*_*MLE*_**	0.0399	0.0418	0.0345	0.0498
	*H*_*Z*_**	0.0472		0.8478	0.9290
	*H*_*CS*_**	0.0480			0.9344
	*H*_*Chao*_**	0.0479			

**Figure 5:**
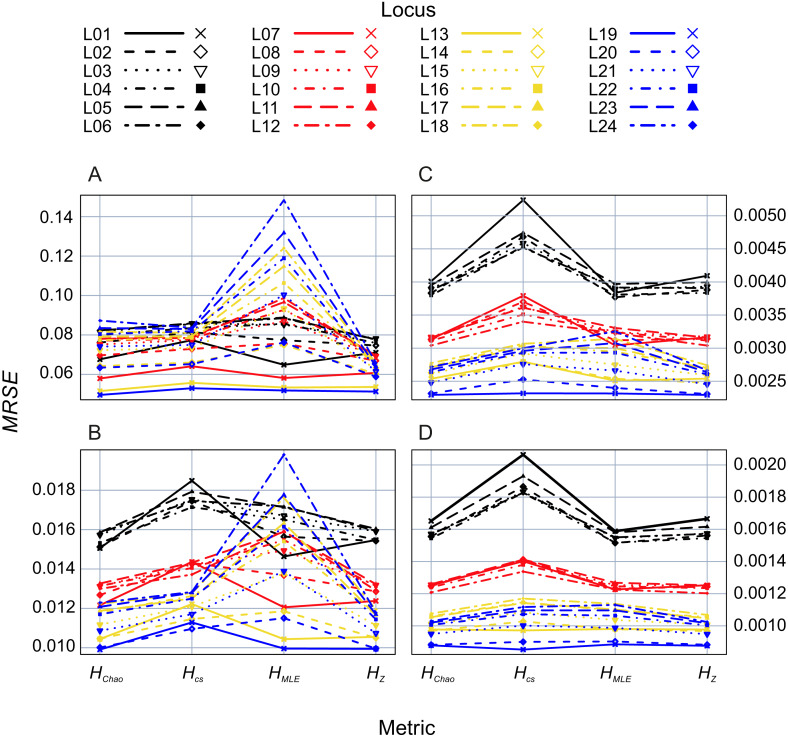
GLM results: locus (L01–L24) effects on mean relative squared error (*MRSE*) of the four Shannon *H* estimators at four sample sizes (A) *Ns* = 5, (B) *Ns* = 20, (C) *Ns* = 80 and (D) *Ns* = 200.

In the second analysis, locus properties were tested. ANOVA of the GLM results showed that locus predictor was significantly associated with the *MRSE* (*p* = 10^−15^) in all metrics. The size of the effect depended on mutation rates, the maximum number of alleles (Fig. 5) and expected heterozygosity at a given locus (Figs. S1–S4). Mutation rates had a more substantial effect on *MRSE* (line colors in Fig. 5) than the maximum number of allelic states at a locus (line types in Fig. 5; Table S1), except for *H*_*MLE*_ at the smallest sample size in which case the error increased at fast mutating loci with the number of allelic states possible (L13-L24). The error level of *H*_*Z*_, *H*_*Chao*_ and *H*_*MLE*_ decreased with the total population’s *He* of the locus (Figs. S1–S4). The slope of this relation was steeper in loci with fewer allelic states allowed (e.g., L01, L07, L13 and L19). In case of *H*_*CS*_ the MRSE at the smallest sample size, *Ns* = 5, was positively correlated with *He* at fast mutating loci with more allelic states allowed, while at the remaining sample sizes the relation was negative.

## Discussion

The problem of sample depencence of genetic diversity measures has been observed in estimation the number of alleles in populations; to tackle the issue, the rarefaction method was proposed for estimating allelic richness instead of the plain number of alleles (El Mousadik & Petit, 1996; Kalinowski, 2004). Allelic richness quickly gained attention and became a popular estimator of genetic variation. On the other hand, the advances in Shannon diversity index estimation proposed by Zahl (1977), Chao & Shen (2003) and Chao, Wang & Jost (2013) remain unnoticed in population genetics studies.

**Table 4:** Minimum, maximum and mean values of the relative bias (*rB*) of all Shannon *H* estimators and sample sizes tested.

Sample size		*H*_*MLE*_**	*H*_*Z*_**	*H*_*CS*_**	*H*_*Chao*_**
	min	−0.3222	−0.2097	NA	−0.2232
5	median	−0.1872	−0.0229	NA	−0.0186
	max	−0.0073	0.1599	NA	0.1344
	min	−0.1252	−0.0939	−0.1063	−0.0956
20	median	−0.0508	−0.0024	−0.0142	−0.0041
	max	0.0335	0.0809	0.0808	0.0777
	min	−0.0527	−0.0423	−0.0420	−0.0424
80	median	−0.0139	−0.0009	−0.0059	−0.0013
	max	0.0258	0.0403	0.0478	0,0407
	min	−0.0318	−0.0259	−0.0222	−0.0261
200	median	−0.0058	−0.0004	−0.0021	−0.0005
	max	0.0185	0.0250	0.0296	0.0248

**Table 5:** Compact letter display of Tukey HSD *post hoc* test of all pair-wise comparisons between the effects of the *H* estimators on *MRSE*. Four independent tests were performed on data with fixed sample sizes.

Sample size	Estimator			
	*H*_*MLE*_**	*H*_*Z*_**	*H*_*CS*_**	*H*_*Chao*_**
5	d	a	b	c
20	d	a	c	b
80	d	a	c	b
200	c	a	b	a

The results of the present study confirm what has been known from species diversity studies (Pielou, 1966), that the original Shannon index, *H*_*MLE*_ is strongly dependent on sample size. This phenomenon is stronger both in more genetically variable populations and in more variable loci, particularly at small sample sizes. It is not possible to estimate the true value of Shannon index using *H*_*MLE*_ when the sample size is small. The most likely explanation for it is that in small samples, the probability that all alleles have been captured is lower than when the sample is large. The so-called *nearly unbiased* estimators also showed some level of bias, even in samples as big as 200 genotypes; however, the difference was negligible (less than 1‰), and the parametric values were well within the 95% confidence intervals of results from the simulated samples. Those measures performed better at more diverse loci and populations, where both *rB* and *MRSE* were, on average, smaller. On the other hand, the error levels of unbiased metrics were more dependent on mutation rates rather than the maximum possible number of allelic states at the locus, which may suggest that the occurrence of low-frequency alleles stemming from numerous mutation events has a stabilising effect on those estimators. Using *H*_*CS*_ bears a high risk of encountering problems when the sample sizes are small, and the level of genetic variation is high, which makes this metric hardly useful in population genetics studies. The performance of this metric is similar to that of the other two unbiased *H* estimators; however, it proved to be less precise than *H*_*Chao*_ and *H*_*Z*_. The analysis of the simulations results suggest the Zahl jackknife estimator *H*_*Z*_ as the most suitable estimator of Shannon diversity index to describe variation at multiallelic loci such as microsatellites. Among the three unbiased estimators, *H*_*Z*_ had the lowest sampling variance and the smallest bias, which also results in the lowest error as compared to the other metrics. For this reason, *H*_*Z*_ should replace traditionally used *H*_*MLE*_ in population genetics studies using microsatellites.

Although all the results presented here were derived from simulating microsatellite loci, the pattern of differences among them shows that the estimates of the Shannon index for less variable loci are more error-prone than for multi-allelic markers. However, as SNP markers are mostly biallelic and the *H* estimates might be more affected by error at individual loci, the effect of the errors averaged over a large number of loci usually used in SNP-based studies may become negligible. Further simulation and empirical tests are necessary to investigate the performance of the Shannon index in SNP loci. While the cost NGS analyses has dropped significantly in recent years, and the present computational power enables analyses of a large amount of data, the problem of small sample sizes in genomic studies remains critical in studies of vulnerable or elusive species, i.e., the cases where the Shannon index is still widely used.

##  Supplemental Information

10.7717/peerj.9391/supp-1 Table S1 The number of failed attempts to estimate HCS at 24 simulated loci (L01-L24) for the sample of Ns = 5 genotypes PC and P500 - two demographic scenarios at which the problems were detected.

10.7717/peerj.9391/supp-2 Table S2 Tukey’s HSD *post-hoc* test of GLM results of locus dependence of MRSE, summarised as compact letter display. The tests were performed independently for each sample size/estimator combination (columns) Letters from *a* to *m* represent ranges of the overappling confidence intervals.

10.7717/peerj.9391/supp-3 Figure S1 GLM results: effects of Nei’s diversity (*Hs*) on mean relative squared error (*MRSE*) of four *H* estimators at the 24 simulated loci *Ns* = 5 genotypes

10.7717/peerj.9391/supp-4 Figure S2 GLM results: effects of Nei’s diversity (*Hs*) on mean relative squared error (*MRSE*) of four *H* estimators at the 24 simulated loci *Ns* = 20 genotypes

10.7717/peerj.9391/supp-5 Figure S3 GLM results: effects of Nei’s diversity (*Hs*) on mean relative squared error (*MRSE*) of four *H* estimators at the 24 simulated loci *Ns* = 80 genotypes

10.7717/peerj.9391/supp-6 Figure S4 GLM results: effects of Nei’s diversity (*Hs*) on mean relative squared error (*MRSE*) of four *H* estimators at the 24 simulated loci *Ns* = 200 genotypes

10.7717/peerj.9391/supp-7 Supplemental Information 7 R code

## Additional Information and Declarations

### Data Availability

The following information was supplied regarding data availability: The code used for simulations is available as a Supplemental File.
