# Supplementary material for: Shannon diversity index: a call to replace the original Shannon’s formula with unbiased estimator in the population genetics studies
Source: PeerJ. 2020 Jun 29;8:e9391. doi: 10.7717/peerj.9391 (PMC7331625; doi:10.7717/peerj.9391)
Supplement: Table S1 — PC and P500 - two demographic scenarios at which the problems were detected. [file peerj-08-9391-s001.docx]

|  | *P_C_* | *P_500_* | Max. no. of allelic states | Mutation rate | |
| --- | --- | --- | --- | --- | --- |
| L01 | 0 | 0 | 3 | 10^-4^ |  |
| L02 | 0 | 0 | 6 | 10^-4^ |  |
| L03 | 0 | 0 | 9 | 10^-4^ |  |
| L04 | 1 | 0 | 12 | 10^-4^ |  |
| L05 | 1 | 1 | 15 | 10^-4^ |  |
| L06 | 0 | 0 | 20 | 10^-4^ |  |
| L07 | 0 | 0 | 3 | 2*10^-4^ |  |
| L08 | 0 | 0 | 6 | 2*10^-4^ |  |
| L09 | 0 | 0 | 9 | 2*10^-4^ |  |
| L10 | 2 | 1 | 12 | 2*10^-4^ |  |
| L11 | 15 | 5 | 15 | 2*10^-4^ |  |
| L12 | 28 | 13 | 20 | 2*10^-4^ |  |
| L13 | 0 | 0 | 3 | 5*10^-4^ |  |
| L14 | 0 | 0 | 6 | 5*10^-4^ |  |
| L15 | 0 | 0 | 9 | 5*10^-4^ |  |
| L16 | 67 | 33 | 12 | 5*10^-4^ |  |
| L17 | 160 | 78 | 15 | 5*10^-4^ |  |
| L18 | 322 | 166 | 20 | 5*10^-4^ |  |
| L19 | 0 | 0 | 3 | 10^-3^ |  |
| L20 | 0 | 0 | 6 | 10^-3^ |  |
| L21 | 0 | 0 | 9 | 10^-3^ |  |
| L22 | 229 | 108 | 12 | 10^-3^ |  |
| L23 | 457 | 259 | 15 | 10^-3^ |  |
| L24 | 673 | 491 | 20 | 10^-3^ |  |
