# Supplementary material for: Shannon diversity index: a call to replace the original Shannon’s formula with unbiased estimator in the population genetics studies
Source: PeerJ. 2020 Jun 29;8:e9391. doi: 10.7717/peerj.9391 (PMC7331625; doi:10.7717/peerj.9391)
Supplement: Table S2 — Letters from a to m represent ranges of the overappling confidence intervals. [file peerj-08-9391-s002.docx]

Table S1. Tukey HSD method analysis of the effects of 24 loci on the relative error of Shannon *H* estimators. Compact letter display - the letters from “*a”* to “*m”* indicate confidence intervals of the locus effects (“*a”* - the smallest and *“m”* - the largest values). All the compared mean values were negative.

| Locus | Mutation  rate | Max. no. of  allelic states |  | *H_Ch_* | | | |  | *H_CS_* | | | |  | *H_MLE_* | | | | *H_Z_* | | | | |  |
| --- | --- | --- | --- | --- | --- | --- | --- | --- | --- | --- | --- | --- | --- | --- | --- | --- | --- | --- | --- | --- | --- | --- | --- |
|  |  |  |  | 5 | 20 | 80 | 200 |  | 5 | 20 | 80 | 200 |  | 5 | 20 | 80 | 200 |  | 5 | 20 | 80 | 200 |  |
| L01 | 0,0001 | 3 |  | d | j | f | e |  | cef | h | h | h |  | c | cd | g | i |  | ij | h | f | e |  |
| L02 | 0,0001 | 6 |  | fhi | j | f | e |  | fgh | h | gh | h |  | d | dfg | g | i |  | jk | h | f | e |  |
| L03 | 0,0001 | 9 |  | km | j | f | e |  | h | h | gh | h |  | e | ghj | g | i |  | k | h | f | e |  |
| L04 | 0,0001 | 12 |  | ijkm | j | f | e |  | gh | h | g | h |  | ef | fhi | g | i |  | k | h | f | e |  |
| L05 | 0,0001 | 15 |  | lm | j | f | e |  | h | h | gh | h |  | f | hj | g | i |  | k | h | f | e |  |
| L06 | 0,0001 | 20 |  | lm | j | f | e |  | h | h | g | h |  | f | hj | g | i |  | k | h | f | e |  |
|  |  |  |  |  |  |  |  |  |  |  |  |  |  |  |  |  |  |  |  |  |  |  |  |
| L07 | 0,0002 | 3 |  | b | efi | e | d |  | b | g | f | g |  | b | b | def | fgh |  | bd | eg | e | d |  |
| L08 | 0,0002 | 6 |  | de | fi | e | d |  | c | g | f | g |  | d | c | def | gh |  | gh | fg | e | d |  |
| L09 | 0,0002 | 9 |  | fh | hi | e | d |  | cef | fg | f | g |  | ef | de | ef | gh |  | hi | g | e | d |  |
| L10 | 0,0002 | 12 |  | ghj | hi | e | d |  | cfg | eg | ef | g |  | g | df | ef | gh |  | hi | g | e | d |  |
| L11 | 0,0002 | 15 |  | ghk | i | e | d |  | eg | g | f | g |  | hi | efg | f | h |  | hi | g | e | d |  |
| L12 | 0,0002 | 20 |  | hk | ghi | de | d |  | deg | deg | df | fg |  | i | efg | def | fgh |  | hi | fg | de | d |  |
|  |  |  |  |  |  |  |  |  |  |  |  |  |  |  |  |  |  |  |  |  |  |  |  |
| L13 | 0,0005 | 3 |  | a | abc | abc | ac |  | a | acd | bc | ac |  | a | a | ab | ac |  | a | ab | ac | ac |  |
| L14 | 0,0005 | 6 |  | c | abc | abc | ac |  | b | ac | bc | bce |  | d | b | ab | ac |  | bc | ab | ac | ac |  |
| L15 | 0,0005 | 9 |  | ef | cde | bc | bc |  | cd | ac | c | ce |  | gh | c | bc | ce |  | cdf | bcd | bc | bc |  |
| L16 | 0,0005 | 12 |  | ghj | df | bd | bc |  | deg | bcef | cd | de |  | j | df | ce | de |  | efg | ce | bc | bc |  |
| L17 | 0,0005 | 15 |  | hkl | dfg | bc | bc |  | eg | bce | cd | de |  | k | fh | cf | ef |  | efg | ce | bc | bc |  |
| L18 | 0,0005 | 20 |  | km | efi | cd | c |  | fgh | bcg | cde | ef |  | l | ij | def | eg |  | fh | def | cd | c |  |
|  |  |  |  |  |  |  |  |  |  |  |  |  |  |  |  |  |  |  |  |  |  |  |  |
| L19 | 0,001 | 3 |  | a | a | a | a |  | a | ab | a | a |  | a | a | a | a |  | a | a | a | a |  |
| L20 | 0,001 | 6 |  | c | ab | a | a |  | b | a | ab | ab |  | d | b | a | ab |  | b | a | a | a |  |
| L21 | 0,001 | 9 |  | fg | bd | ab | ab |  | ce | ac | bc | bcd |  | i | c | b | bcd |  | bde | ac | ab | ab |  |
| L22 | 0,001 | 12 |  | jkm | df | bc | bc |  | fgh | bce | c | ce |  | k | fh | cd | ce |  | cdfg | bce | bc | bc |  |
| L23 | 0,001 | 15 |  | mn | efh | bc | bc |  | fgh | cg | cd | ce |  | m | j | def | de |  | dfg | ce | bc | bc |  |
| L24 | 0,001 | 20 |  | n | fi | bc | bc |  | gh | cg | cd | de |  | n | k | f | eg |  | fh | cef | bc | bc |  |
