# Supplementary figures and images for: Shannon diversity index: a call to replace the original Shannon’s formula with unbiased estimator in the population genetics studies
Source: PeerJ. 2020 Jun 29;8:e9391. doi: 10.7717/peerj.9391 (PMC7331625; doi:10.7717/peerj.9391)

$H_{MLE}$      $H_Z$      $H_{CS}$      $H_{Chao}$ 
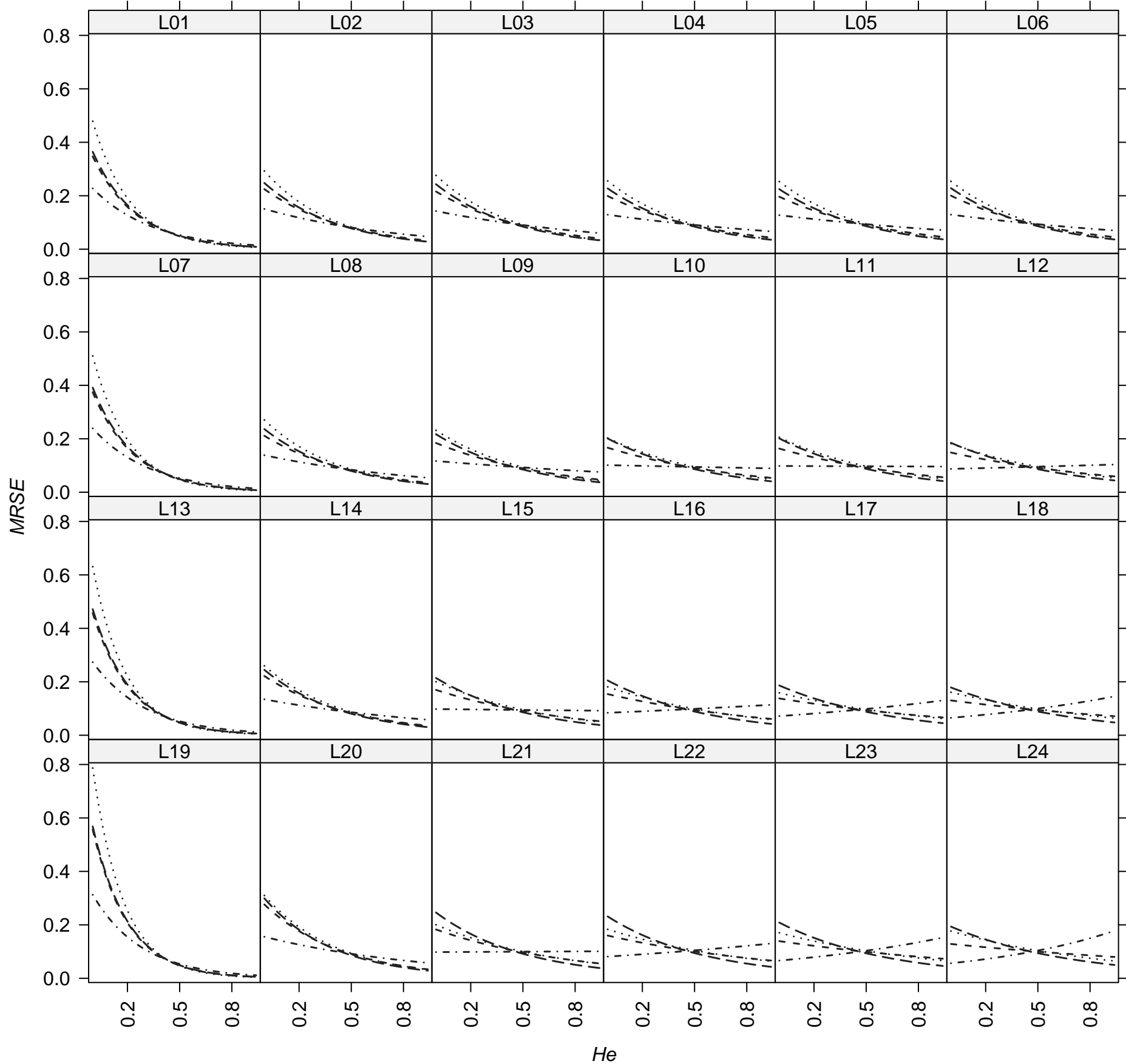

Supplement: Figure S1 [file peerj-08-9391-s003.pdf]

$H_{MLE}$     $H_Z$     $H_{CS}$     $H_{Chao}$ 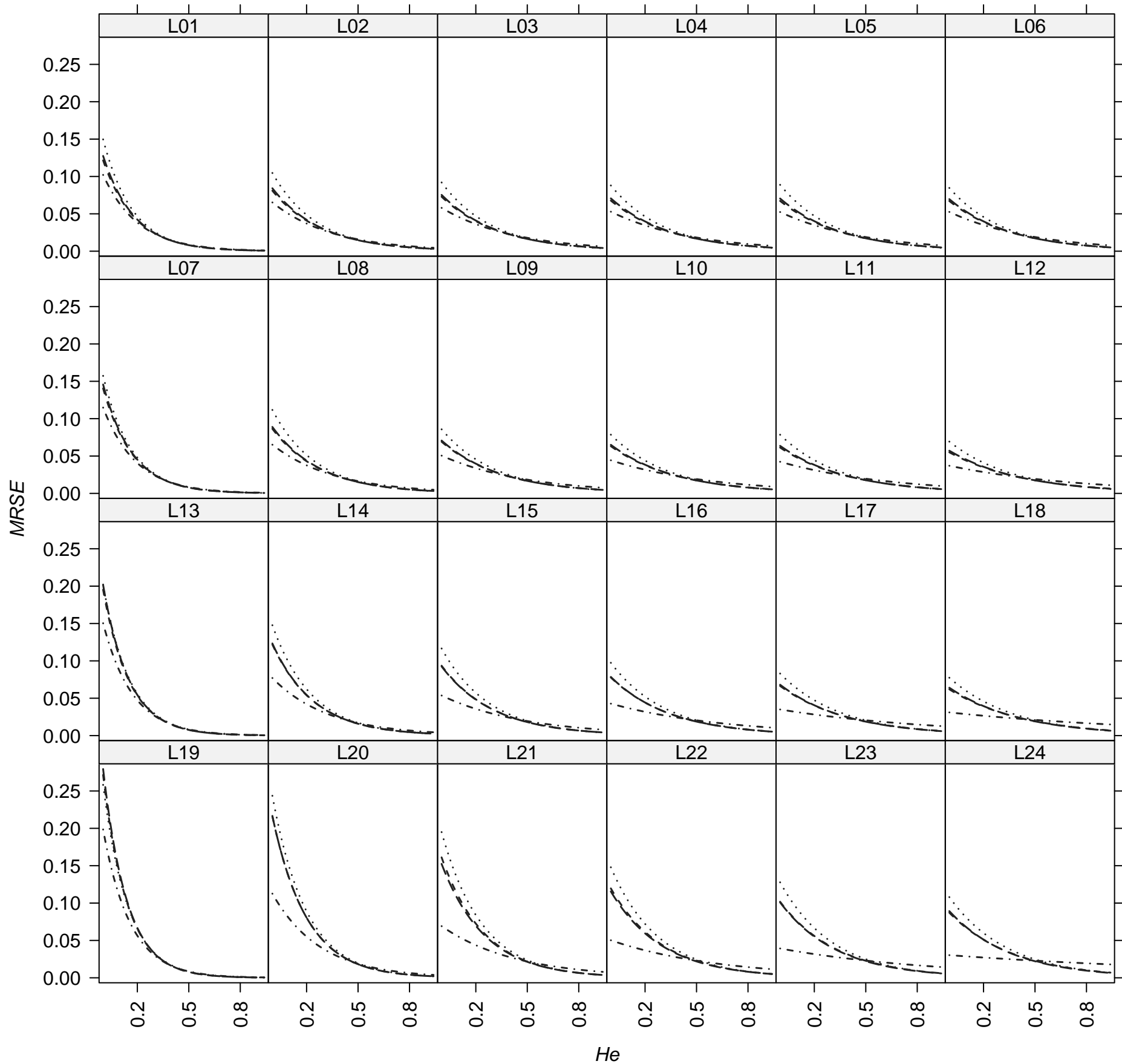

Supplement: Figure S2 [file peerj-08-9391-s004.pdf]

$H_{MLE}$     $H_Z$     $H_{CS}$     $H_{Chao}$ 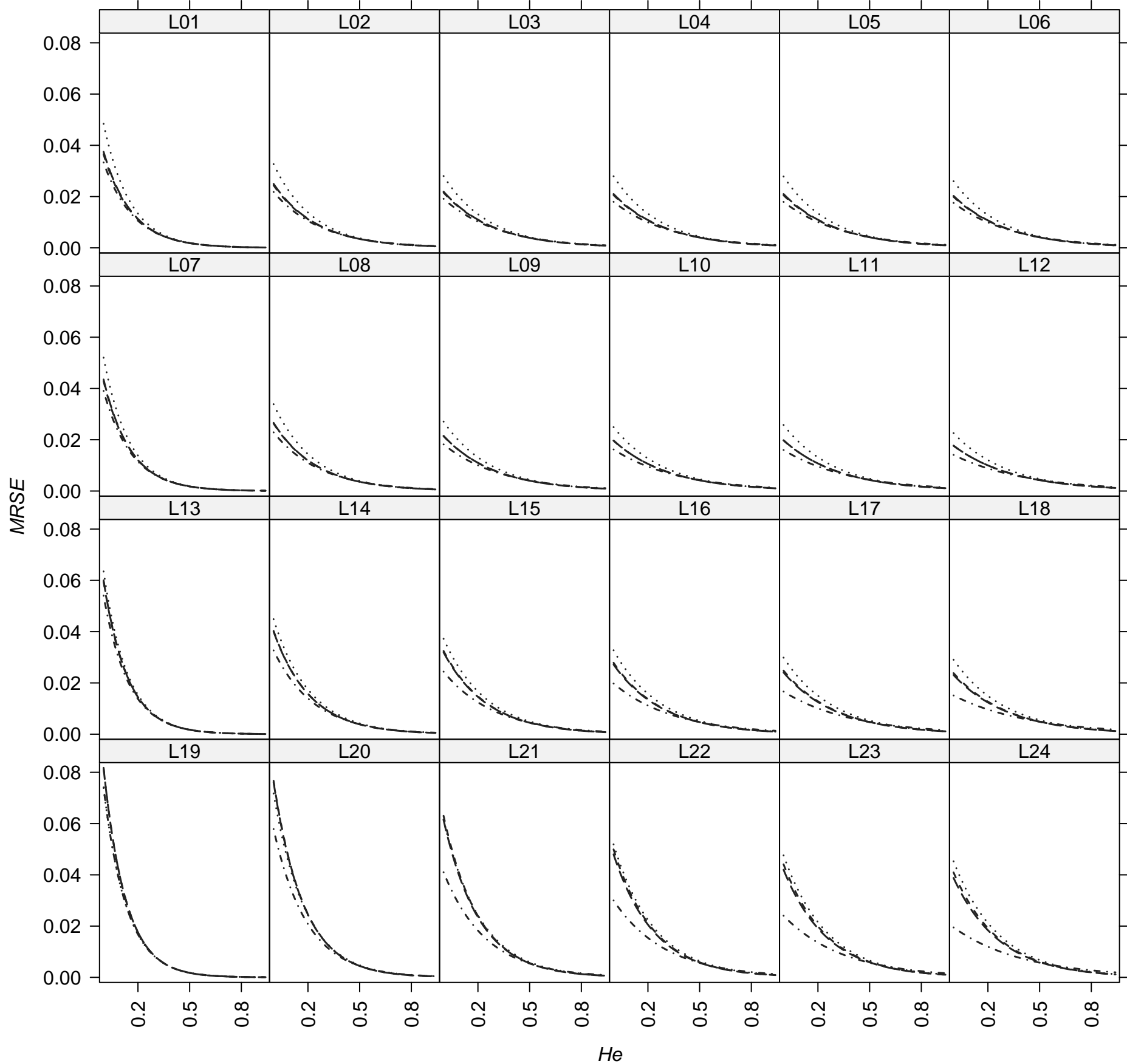

Supplement: Figure S3 [file peerj-08-9391-s005.pdf]

200

 $H_{MLE}$      $H_Z$      $H_{CS}$      $H_{Chao}$ 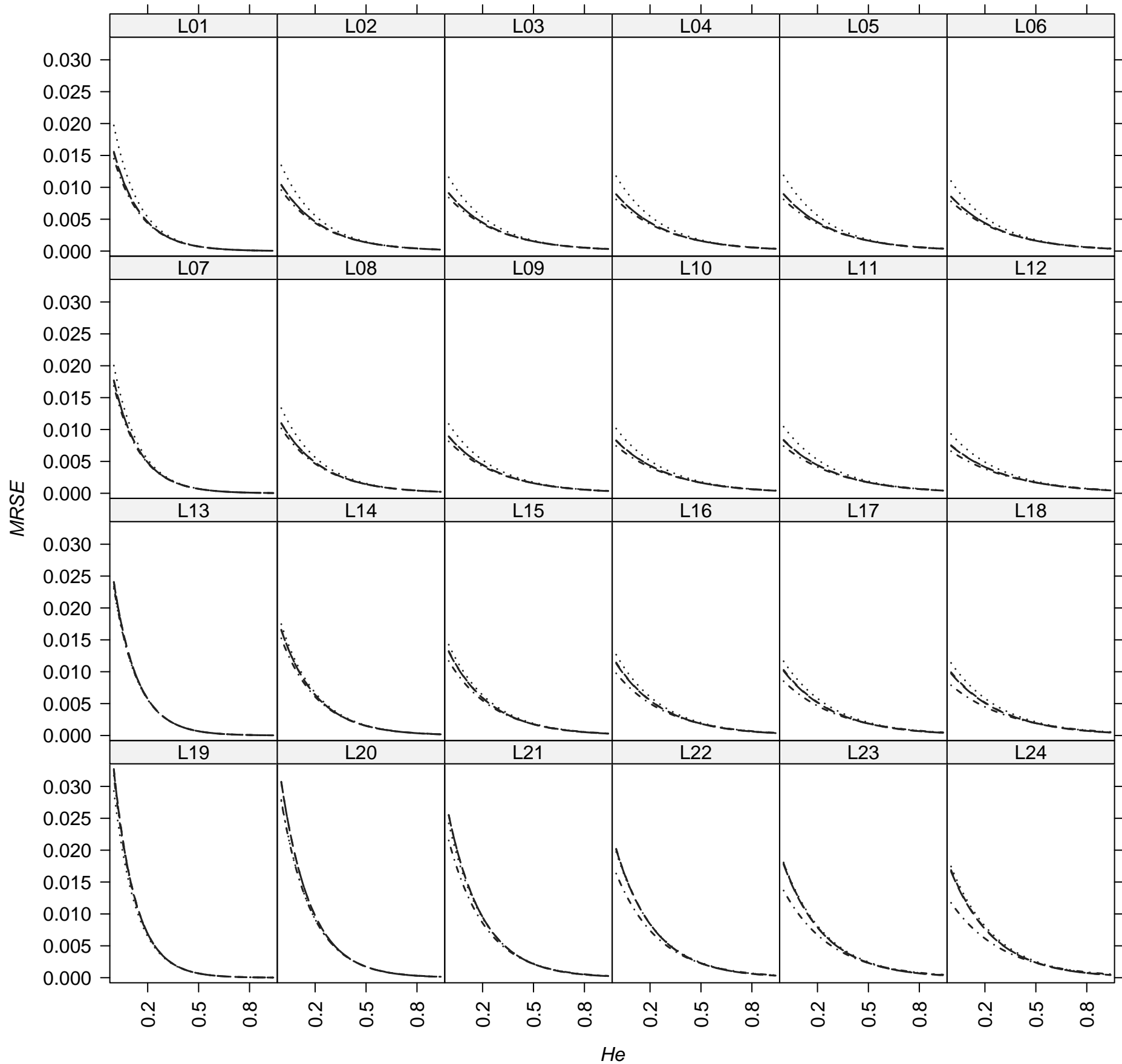

Supplement: Figure S4 [file peerj-08-9391-s006.pdf]
